# Supplementary material for: Peripheral inhibition of IL-6 signaling with tocilizumab improves stroke outcomes in aged mice but requires sex-specific dosing
Source: bioRxiv. 2026 May 6:2026.05.01.722347. Preprint. [Version 1] doi: 10.64898/2026.05.01.722347 (PMC13174368; doi:10.64898/2026.05.01.722347)
Supplement: 1 [file NIHPP2026.05.01.722347V1-supplement-1.pdf]

bioRxiv preprint doi: https://doi.org/10.64898/2026.05.01.722347; this version posted May 6, 2026. The copyright holder for this preprint (which was not certified by peer review) is the author/funder, who has granted bioRxiv a license to display the preprint in perpetuity. It is made available under a CC-BY 4.0 International license.

Supplementary Materials

Materials and Methods

Animals

Aged male and female C57BL/6J mice (18-20 months old; stock #000664, The Jackson Laboratory) were used for all primary experiments. A small preliminary cohort of young male C57BL/6J mice (3-4 months old) was also included. Young mice were purchased at approximately 2 months of age and acclimated for at least 4 weeks prior to experimentation. Aged mice were purchased at 6–9 months of age and maintained in the animal facility until reaching 18–20 months of age.

All animals were housed under specific pathogen-free conditions at McGovern Medical School with ad libitum access to food and water. All experimental procedures were conducted in accordance with NIH guidelines for the care and use of laboratory animals and were approved by the Institutional Animal Care and Use Committee at The University of Texas Health Science Center at Houston. Animals were randomly assigned to surgical and treatment groups.

IL-6R Knockout Mice

Interleukin-6 receptor (IL-6R) knockout mice were generated by crossing IL-6Rα floxed mice (stock #012944, The Jackson Laboratory) with CMV-Cre transgenic mice (stock #006054, The Jackson Laboratory) to achieve germline deletion of IL-6R.

Genotyping was performed by PCR using primers provided by The Jackson Laboratory to detect wild-type, floxed, and recombined IL-6Rα alleles. The following primers were used: wild-type IL-6Rα allele (5′-CTGGGACCCGAGTTACTACTT-3′ and 5′-CAGCAACACCGTGAACTCCTTT-3′), floxed IL-6Rα allele (5′-GCCTGGGTGGAGAGGCTTTTT-3′ and 5′-CCCAGTGAGCTCCACCATCAAA-3′), and recombined IL-6Rα allele (5′-CTGGGACAGGGAAGGGCTTTT-3′ and 5′-CCCAGTGAGCTCCACCATCAAA-3′).

Experimental knockout mice were identified by the absence of both wild-type and floxed IL-6Rα alleles and the presence of the recombined allele. Because recombination occurs in the germline, the CMV-Cre transgene was not maintained in experimental animals to avoid potential Cre-related effects.

Transient Stroke Model

Mice were randomly assigned to stroke or sham surgery and further subdivided into treatment groups, resulting in four groups per cohort. Transient cerebral ischemia was induced by 60 minutes of reversible middle cerebral artery occlusion (MCAO) as previously described (7). A 6-0 silicone-coated monofilament (Doccol Corporation, Sharon, MA) was introduced into the right internal carotid artery to occlude the origin of the middle cerebral artery. Following the occlusion period, mice were placed in a temperature-controlled recovery cage for 1 hour prior to reperfusion, at which time the monofilament was withdrawn to restore cerebral blood flow. Rectal temperature was

bioRxiv preprint doi: https://doi.org/10.64898/2026.05.01.722347; this version posted May 6, 2026. The copyright holder for this preprint (which was not certified by peer review) is the author/funder, who has granted bioRxiv a license to display the preprint in perpetuity. It is made available under a CC-BY 4.0 International license.

continuously monitored throughout MCAO and reperfusion and maintained at 37°C. Sham-operated mice underwent identical procedures except that the monofilament was not advanced to occlude the middle cerebral artery.

Five hours after ischemia onset, mice received a single intraperitoneal injection of tocilizumab (20 mg/kg or 100 mg/kg for the high-dose cohort) or control human IgG (R&D Systems, Minneapolis, MN). Postoperatively, mice received daily subcutaneous injections of sterile saline and wet mash for 7 days following reperfusion. Animals were euthanized at either 3 or 35 days after MCAO

Drug Dosing

For the 35-day cohorts of aged male and female mice and the 3-day cohort of young male mice, animals received a single intraperitoneal injection of tocilizumab or control human IgG 5 hours after ischemia onset at doses of 20 mg/kg or 100 mg/kg.

Behavioral Testing

Behavioral assessments were performed to evaluate neurological, sensorimotor, cognitive, and exploratory outcomes following stroke. Neurological deficit scores were assessed using the Bederson 5-point standardized scoring (34), daily during the first week after surgery (days 1-7) and subsequently on days 10, 14, 20, 28, and 35. Sensorimotor asymmetry was evaluated using the corner test on days 7, 14, and 20 after surgery (48). Working memory and exploratory behavior were assessed using the Y-maze test on days 7 and 21 (49). These time points were selected to minimize repeated testing while enabling longitudinal assessment of recovery.

Spatial learning and memory were assessed using the Barnes maze. Training was conducted on days 27–29 after surgery, and probe testing was performed on day 31. The Barnes maze consisted of an elevated circular platform with 20 equally spaced holes, one of which led to an escape box located beneath the platform. Visual cues were positioned around the testing room to facilitate spatial navigation. At the start of each trial, mice were placed in a central start chamber for 1 minute. After removal of the chamber, mice were allowed to explore the maze for up to 3 minutes to locate the escape hole. If a mouse failed to locate the escape hole within the allotted time, it was guided to the correct location and allowed to remain there for 1 minute. Mice underwent three trials per day during training

Behavioral testing was performed prior to surgery to establish baseline performance and identify animals with preexisting bias. All behavioral testing and analysis were conducted by investigators blinded to treatment group. Behavioral videos were analyzed using EthoVision XT software (Noldus Information Technology, Leesburg, VA).

Cresyl Violet and TTC Staining

Cresyl violet staining was used to quantify infarct volume at 3 days and brain atrophy at 35 days after stroke. Mice were perfused with 1× PBS followed by 4% paraformaldehyde. Brains were post-fixed and cryoprotected in 30% sucrose at 4°C overnight. Coronal sections (30 μm) were cut using a microtome. Eight equally spaced sections per brain, separated by 360 μm, were mounted onto slides and stained with cresyl violet. Images were acquired and analyzed by an investigator blinded to treatment group using SigmaScan Pro software to quantify infarct area and tissue atrophy.

bioRxiv preprint doi: https://doi.org/10.64898/2026.05.01.722347; this version posted May 6, 2026. The copyright holder for this preprint (which was not certified by peer review) is the author/funder, who has granted bioRxiv a license to display the preprint in perpetuity. It is made available under a CC-BY 4.0 International license.

For 2,3,5-triphenyltetrazolium chloride (TTC) staining, animals were euthanized and brains were rapidly removed and briefly chilled at −80°C for 4 minutes to facilitate sectioning. Brains were cut into five 2-mm coronal sections spanning from the olfactory bulb to the cerebellum. Sections were incubated in 1.5% TTC (Sigma, St. Louis, MO) and subsequently fixed in 4% formalin. Digital images were acquired, and infarct area was quantified using SigmaScan Pro software.

Gut Permeability Assessment

Intestinal permeability was assessed 3 days after surgery using fluorescein isothiocyanate–dextran (FITC–dextran, 4 kDa). FITC–dextran was prepared at 50 mg/mL in sterile water. On day 3 after surgery, mice were fasted for 5 hours prior to testing and then administered FITC–dextran by oral gavage at a dose of 6 mg per 10 g body weight. One hour after gavage, mice were euthanized and blood was collected by cardiac puncture using heparinized syringes. Blood samples were centrifuged at 14,000 rpm for 14 minutes to isolate plasma. Plasma FITC–dextran concentrations were measured using an EnSpire plate reader (PerkinElmer) with excitation at 470 nm and emission at 520 nm. A standard curve was generated using serial dilutions of FITC–dextran, along with blank controls.

Lung Bacterial Colony-Forming Unit (CFU) Measurement

Pulmonary bacterial burden was assessed by quantifying colony-forming units (CFUs) in lung homogenates at 35 days after surgery. Following euthanasia, lungs were collected, weighed, and homogenized in sterile 1% saponin in 1× PBS at a concentration of 50 mg tissue per mL. An aliquot of 50 µL of homogenate (corresponding to approximately 2.5 mg of lung tissue) was plated onto 5% sheep blood agar plates (Carolina Biological Supply, Burlington, NC) and incubated at 37°C for 16 hours. After incubation, bacterial colonies were counted. CFU values were normalized to tissue weight and expressed as CFUs per mL of homogenate.

MRI Acquisition and Analysis

Magnetic resonance imaging (MRI) was performed using a Bruker 7T Avance system equipped with a 20-cm bore, microgradients, and ParaVision 5.1 software. Brain images were acquired using a rapid acquisition with relaxation enhancement (RARE) sequence with the following parameters: repetition time (TR) 2500 ms, echo time (TE) 36 ms, and number of averages (NA) 2. The field of view was 3 cm with a matrix size of 256 × 256 and a slice thickness of 500 µm. MRI analyses were performed in mice treated with the 100 mg/kg dose of tocilizumab.

Assessment of Cerebrospinal Fluid and Ventricular Volume

Following transcardial perfusion with PBS followed by 4% paraformaldehyde (PFA), brains were harvested with the skull intact. Skin, muscle, ears, nasal tip, and lower jaw were removed to expose the skull. The intact head was fixed in 4% PFA at 4°C and subsequently transferred to 40 mL of 0.01% sodium azide in PBS with gentle agitation for 7 days at 4°C. Samples were then incubated in a contrast solution containing 5 mM gadopentetate dimeglumine (Bayer HealthCare Pharmaceuticals Inc., Wayne, NJ) and 0.01% sodium azide in PBS for 21 days at 4°C to enhance MRI contrast.

bioRxiv preprint doi: https://doi.org/10.64898/2026.05.01.722347; this version posted May 6, 2026. The copyright holder for this preprint (which was not certified by peer review) is the author/funder, who has granted bioRxiv a license to display the preprint in perpetuity. It is made available under a CC-BY 4.0 International license.

MRI datasets were analyzed using OsiriX MD software. DICOM images were imported, and whole-brain segmentation was performed. Regions corresponding to cerebrospinal fluid (CSF) and ventricular spaces were identified on each slice. Ventricular and CSF areas ipsilateral and contralateral to the injury were manually delineated and quantified to assess brain atrophy and ventricular enlargement. All MRI segmentation and analyses were performed by investigators blinded to treatment group. Assessment of cerebrospinal fluid and ventricular volume.

ELISA

Plasma levels of IL-6, soluble IL-6 receptor (sIL-6R), and ADAM17 in human samples were measured using commercially available ELISA kits (R&D Systems, D6050B; Abcam, ab46029; and Invitrogen, EHADAM17, respectively) according to the manufacturers' instructions.

For mouse plasma and neutrophil culture supernatants, IL-6 and sIL-6R levels were measured using ELISA kits from R&D Systems (DY008 and DY1830) according to the manufacturers' protocols. Samples and standards were processed as specified by each kit, and absorbance was measured using a microplate reader.

Ex-vivo Neutrophil Experiments

Bone marrow neutrophils were isolated from mouse femurs following perfusion. Femurs were dissected, cleared of surrounding tissue, briefly sterilized in 70% ethanol, and rinsed in 1× PBS. Bone marrow cells were collected by flushing the femurs with 5 mL Hanks' Balanced Salt Solution (HBSS) without calcium or magnesium, supplemented with 2% heat-inactivated fetal bovine serum (FBS).

The cell suspension was filtered through a 40 μm cell strainer into a 50 mL conical tube and washed twice with HBSS containing 2% FBS. Cells were resuspended and layered onto a discontinuous Histopaque gradient (Histopaque 1.077 and 1.119; Sigma). Samples were centrifuged at 500 × g for 30 minutes at room temperature with no brake. Mononuclear cells at the HBSS–Histopaque 1.077 interface were discarded, and neutrophils were collected from the interface between Histopaque 1.077 and 1.119. Cells were washed twice with HBSS containing 2% FBS and further purified using a Mouse Neutrophil Isolation Kit (STEMCELL Technologies, catalog #19762) according to the manufacturer's instructions. Neutrophil purity was routinely >90%, as assessed by morphological criteria and differential cell counts.

Isolated neutrophils were plated in 6-well plates and treated with the pan-caspase inhibitor Q-VD-OPh (MP Biomedicals) or vehicle control. Cells were stimulated with 1 μM N-formyl-methionyl-leucyl-phenylalanine (fMLP; Sigma-Aldrich) for 1 hour. Supernatants and cell lysates were collected for ELISA and qPCR analyses.

Oxygen Glucose Deprivation (OGD) and Flow Cytometry

To model ischemia-reperfusion conditions *in vitro*, splenocytes isolated from male and female mice were subjected to oxygen–glucose deprivation (OGD) as previously described (50). Mice were euthanized by intraperitoneal Avertin injection and transcardially perfused with 20 mL cold sterile PBS. Spleens were harvested under sterile conditions and mechanically dissociated through a 70 μm cell strainer to generate a single-cell suspension. Red blood cells were lysed using Tris-ammonium chloride buffer (STEMCELL Technologies) for 10 minutes. Cells were then washed and resuspended in culture medium.

bioRxiv preprint doi: https://doi.org/10.64898/2026.05.01.722347; this version posted May 6, 2026. The copyright holder for this preprint (which was not certified by peer review) is the author/funder, who has granted bioRxiv a license to display the preprint in perpetuity. It is made available under a CC-BY 4.0 International license.

For OGD exposure, culture medium was replaced with serum-free, glucose-free Locke's buffer (154 mM NaCl, 5.6 mM KCl, 2.3 mM $CaCl_2$, 1 mM $MgCl_2$, 3.6 mM $NaHCO_3$, 5 mM HEPES, and 5 mg/mL gentamicin; pH 7.2). Cells were placed in a hypoxia chamber maintained at 95% N_2 and 5% CO_2 for 1 hour. Control cells were maintained under normoxic conditions in glucose-containing medium at 95% air and 5% CO_2. Following OGD, splenocytes were treated with the pan-caspase inhibitor Q-VD-OPh (0.05 mM) or vehicle control for 1 hour prior to flow cytometry staining.

Cells were stained with Zombie Aqua viability dye (BioLegend) and incubated with Fc receptor blocking reagent (BioLegend) prior to staining with fluorophore-conjugated antibodies: CD45-R718, CD126 (IL-6R)-PE, Ly6G-eFluor450, CD11b-APC, and F4/80-PE-Cy7. Data were acquired using a CytoFLEX S (Beckman Coulter) or FACSMelody (BD Biosciences) and analyzed with FlowJo software (BD Biosciences). Myeloid populations were identified by sequential gating of $CD45^+$ leukocytes followed by $CD11b^+$ cells. Neutrophils were defined as $CD11b^+Ly6G^+$ cells, and macrophages as $CD11b^+F4/80^+$ cells. A minimum of 500,000 events were collected per sample. Fluorescence-minus-one (FMO) controls, tissue-matched controls, and unstained controls were used to establish gating thresholds.

Statistical Analysis

Data are presented as mean ± SEM with all individual data points shown, unless otherwise specified. Two-group comparisons were performed using unpaired t-tests with Welch's correction. Comparisons involving more than two groups were analyzed by two-way ANOVA with Sidak's multiple comparisons test. These analyses were applied to behavioral outcomes, brain atrophy measurements, ELISA assays (IL-6R, IL-6, LBP), and lung bacterial colony-forming unit (CFU) counts.

Survival data were analyzed using Kaplan–Meier survival curves with comparisons by the Mantel–Cox log-rank test. Neurological deficit scores (NDS) are presented as median (interquartile range) and were analyzed using two-way ANOVA with Sidak's multiple comparisons test. Statistical significance was defined as $p < 0.05$. All mouse data analyses were performed using GraphPad Prism 9.

For human biomarker analyses, demographic variables were compared between sexes using the Wilcoxon rank-sum test for continuous variables and chi-squared or Fisher's exact tests for categorical variables. Biomarker concentrations for IL-6, soluble IL-6 receptor (sIL-6R), and ADAM17 were log-transformed prior to analysis due to skewed distributions. Univariable comparisons were performed using two-sample t-tests. Multivariable linear regression models were used to assess associations between sex and biomarker levels, adjusting for age and NIH Stroke Scale (NIHSS) score. Human data analyses were conducted using R (version 4.5.2; R Foundation for Statistical Computing, Vienna, Austria).

Patient Samples

Patients with acute ischemic stroke, with confirmed images, admitted to Memorial Hermann Hospital (Houston, TX, USA) were enrolled in this study. All participants or their surrogates provided written or verbal informed consent (IRB- HSC-MS-17-0452). Plasma samples were collected 24 hours after the last known normal. All the patients had a MCAO stroke of more than 25% of MCA territory, and no hemorrhagic transformation. Exclusion criteria for all participants are recent blood transfusions, chronic steroid or immunosuppressant use, pregnancy, and active dialysis treatment. Detailed patient characteristics are provided in Supplementary Table 1A.

Peripheral inhibition of IL-6 signaling with tocilizumab improves stroke outcomes in aged mice but requires sex-specific dosing

Department of Neuroscience, University of Connecticut School of Medicine, Farmington, CT , CT 06030, USA. [2]Department of Neurology, McGovern Medical School, University of Texas Health Science Center at Houston, Houston, TX 77030, USA. [3]Department of Orthopedic Surgery, Yale School of Medicine, New Haven, CT, 06510, USA

A

	IL-6 (n=127)			sIL-6R (n=163)			ADAM 17 (n=99)		
	Female (n=56)	Male (n=71)	p-value	Female (n=82)	Male (n=81)	p-value	Female (n=45)	Male (n=54)	p-value
**Age, median [IQR]**	72.50 [65.00, 84.00]	67.00 [59.00, 75.00]	0.007	73.00 [63.00, 84.00]	66.00 [57.00, 75.00]	0.001	72.00 [65.00, 83.00]	68.00 [59.00, 75.00]	0.038
**Race, n (%)**			0.627			0.039			0.349
**- Asian**	1 (1.79%)	3 (4.23%)		4 (4.88%)	5 (6.17%)		0 (0.0%)	2 (3.70%)	
**- Black or African American**	12 (21.43%)	20 (28.17%)		18 (21.95%)	34 (41.98%)		8 (17.78%)	16 (29.63%)	
**- Hispanic**	1 (1.79%)	3 (4.23%)		1 (1.22%)	0 (0.0%)		1 (2.22%)	2 (3.70%)	
**- Other**	9 (16.07%)	11 (15.49%)		15 (18.29%)	15 (18.52%)		8 (17.78%)	9 (16.67%)	
**- Unknown or not reported**	0 (0.0%)	1 (1.41%)		0 (0.0%)	0 (0.0%)		0 (0.0%)	0 (0.0%)	
**- White**	33 (58.93%)	33 (46.48%)		44 (53.66%)	27 (33.33%)		28 (62.22%)	25 (46.30%)	
**Hispanic, n (%)**			1			0.726			1
**- Yes**	12 (21.43%)	15 (21.13%)		18 (21.95%)	15 (18.52%)		10 (22.22%)	11 (20.37%)	
**- No**	44 (78.57%)	56 (78.87%)		64 (78.05%)	66 (81.48%)		35 (77.78%)	43 (79.63%)	
**NIHSS, median [IQR]**	16.00 [10.00, 21.00]	13.00 [7.50, 18.00]	0.035	15.50 [10.00, 21.00]	12.00 [7.00, 18.00]	0.021	17.00 [11.00, 21.00]	12.50 [8.00, 19.00]	0.06

IQR, interquartile range

B

	IL-6 (n=127)		sIL-6R (n=163)		ADAM 17 (n=99)	
	Estimate (SE)	p-value	Estimate (SE)	p-value	Estimate (SE)	p-value
**Univariable analysis based on t-test**						
**Sex, Female**	0.403 (0.255)	0.127	0.605 (0.010)	<0.001	-0.366 (0.348)	0.284
**Multivariable analysis based on multivariable linear regression model**						
**Sex, Female**	0.347 (0.266)	0.195	0.598 (0.105)	<0.001	-0.358 (0.359)	0.322
**Age**	0.003 (0.011)	0.783	0.0007 (0.0036)	0.840	-0.013 (0.014)	0.365
**NIHSS**	0.014 (0.017)	0.405	0.0006 (0.0073)	0.931	0.019 (0.022)	0.390

SE, Standard Error; reference group is male; Outcomes were log-transformed

Supplementary Table 1. Patient demographics and analysis of circulating inflammatory markers. (A) Patient demographics stratified by sex.(B) Univariable and multivariable linear regression analyses of log-transformed IL-6, sIL-6R, and ADAM17 levels by sex.

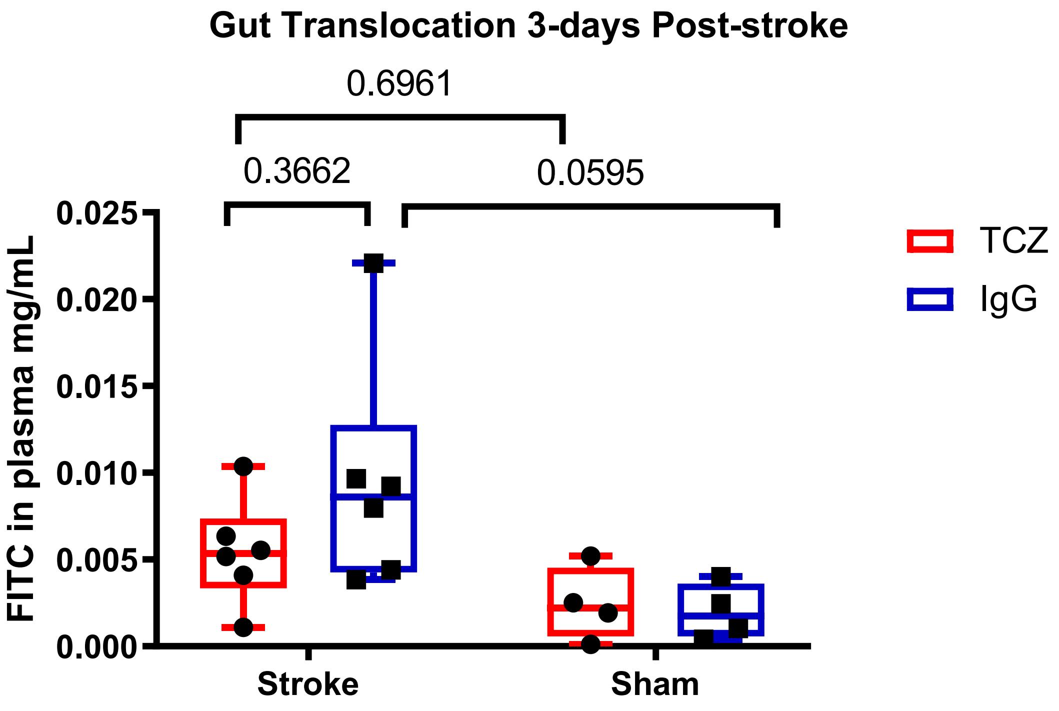

Supplementary Figure 1. Gut translocation 3-days post-stroke. Intestinal permeability was assessed at 3-days post-stroke with fluorescein isothiocyanate-dextran (FITC-dextran), 4 kDa. Mice were fasted for 5-hours and then administered FITC-dextran via oral gavage (6mg/10g pre-stroke body weight) followed by euthanasia 1-hour later for measurement of plasma FITC-dextran concentration (n=4-6). Data presented as mean +/- SEM and analyzed using 2-way ANOVA with Tukey's multiple comparisons test.
